# Entrainment in the master equation

**DOI:** 10.1098/rsos.172157

**Published:** 2018-04-25

**Authors:** Michael Margaliot, Lars Grüne, Thomas Kriecherbauer

**Affiliations:** 1 School of Electrical Engineering and the Sagol School of Neuroscience, Tel-Aviv University, Tel-Aviv 69978, Israel; 2 Mathematical Institute, University of Bayreuth, 95440 Bayreuth, Germany

**Keywords:** cooperative dynamical systems, first integral, stability, contractive systems, Metzler matrix, asymmetric simple exclusion process

## Abstract

The master equation plays an important role in many scientific fields including physics, chemistry, systems biology, physical finance and sociodynamics. We consider the master equation with periodic transition rates. This may represent an external periodic excitation like the 24 h solar day in biological systems or periodic traffic lights in a model of vehicular traffic. Using tools from systems and control theory, we prove that under mild technical conditions every solution of the master equation converges to a periodic solution with the same period as the rates. In other words, the master equation entrains (or phase locks) to periodic excitations. We describe two applications of our theoretical results to important models from statistical mechanics and epidemiology.

## 1. Introduction

Consider a physical system that can be in one of exactly *N* possible configurations and let *x*_*i*_(*t*) denote the probability that the system is in configuration *i* at time *t*. We record the probabilities of all configurations at time *t* by the (column) state-vector $x(t):=\left[\begin{array}{c} x_{1}(t)\\ \vdots \\ x_{N}(t) \end{array}\right]$. Every entry of this vector takes values in the closed interval [0,1].

The *master equation* describes the time evolution of these probabilities. It can be explained intuitively as describing the balance of probability currents going in and out of each possible state. To formulate the master equation for a specific model, one needs to know the rates of transition *p*_*ij*_ from configuration *i* to configuration *j*. A rigorous derivation of the master equation for a chemically reacting gas-phase system that is kept well stirred and in thermal equilibrium is given in [1]. The master equation plays a fundamental role in physics (where it is sometimes referred to as the Pauli master equation), chemistry, systems biology, sociodynamics and more. (For example, see the monographs [2,3] for more details.)

In this paper, we treat the general case where the transition rates *p*_*ij*_ may depend on both time *t* and on the probability distribution *x*(*t*) at time *t*. The resulting system of differential equations (see (1.2) below) constitutes a time-varying nonlinear dynamical system.

Note that even the special case, where the transition rates do not depend on the state *x* (the master equation (1.2) is then linear), is of general interest because it is intimately connected with the theory of Markov processes on finite configuration spaces. The relation is the following: such a Markov process is uniquely determined by an initial probability distribution on the configurations 1,…,*N* and by *transition probabilities* (*Q*_*τ*_(*t*))_*ij*_ that denote the probabilities to be in configuration *i* at time *t* given that the system is in configuration *j* at time *τ*<*t*. These transition probabilities need to satisfy the Chapman–Kolmogorov equations which are essentially equivalent to the condition that the columns of the matrix *Q*_*τ*_(*t*) satisfy the linear version of the master equation known as the *forward equation* in the theory of Markov processes [4,5].

In many physical systems, the number of possible configurations *N* can be very large. For example, the well-known *totally asymmetric simple exclusion principle* TASEP model (e.g. [6,7] and the references therein) includes a lattice of *n* consecutive sites, and each site can be either free or occupied by a particle, so the number of possible configurations is *N*=2^*n*^. In such cases, simulating the master equation and numerically calculating its steady state may be difficult even for small values of *n* and special methods must be applied (e.g. [7,8]).

Here, we are interested in deriving theoretical results that hold for any *N*. Specifically, we consider the case where the transition rates *p*_*ij*_(*t*,*x*) are periodic in time *t* with a common period *T*>0. In this situation, we arrive at a *T*-*periodic master equation*. For such systems, we consider the problem of entrainment (or phase-locking):

**Problem 1.1**

Given a system described by a *T*-periodic master equation, determine if for every initial condition the probabilities *x*_*i*_(*t*), *i*=1,…,*N*, converge to a periodic solution with period *T*. If this is so, determine if the periodic solution is unique or not.

In other words, if we view the transition rates as a *T*-periodic excitation, then the problem is to determine if the state of the system entrains, that is, converges to a periodic trajectory with the same period *T*. If this is so, an important question is whether there exists a unique periodic trajectory *γ* and then every solution converges to *γ*.

Entrainment is important in many natural and artificial systems. For example, organisms are often exposed to periodic excitations like the 24 h solar day and the periodic cell-cycle division process. Proper functioning often requires accurate entrainment of various biological processes to this excitation [9]. For example, cardiac arrhythmia is a heart disorder occurring when every other pulse generated by the sinoatrial node pacemaker is ineffective in driving the ventricular rhythm [10].

Epidemics of infectious diseases often correlate with seasonal changes and the required interventions, such as pulse vaccination, may also need to be periodic [11]. In mathematical population models, this means that the so-called *transmission parameter* is periodic, with a period of 1 year, and entrainment means that the spread of epidemics converges to a periodic pattern with the same period. As another example, traffic flow is often controlled by periodically varying traffic lights. In this context, entrainment means that the traffic flow converges to a periodic pattern with the same period as the traffic lights. This observation could be useful for the study of the *green wave phenomenon* [12]. Another example, from the field of power electronics, involves connecting a synchronous generator to the electric grid. The periodically varying voltage in the grid may be interpreted as a periodic excitation to the generator, and proper functioning requires the generator to entrain to this excitation (e.g. [13] and the references therein).

Our main results provide affirmative answers for problem 1.1 under quite general assumptions. Basic regularity assumptions on the transition probabilities that are required throughout the paper are summarized in assumption 2.1. In theorem 2.2, we then formulate with (2.5), see also (2.3), a condition that guarantees entrainment. It is observed in corollary 2.3 that condition (2.5) is always satisfied in the linear case. Uniqueness of the periodic attractor is shown in theorem 2.5 under the additional assumption of irreducibility, a condition that is well known in the theory of Markov processes (a definition of irreducibility is provided just before the statement of theorem 2.5).

In the special case of time-invariant rates, problem 1.1 reduces to determining if every solution converges to a steady state, and whether there exists a unique steady state. Indeed, time-invariant rates are *T*-periodic for any *T*>0 and thus entrainment means convergence to a solution that is *T*-periodic for any *T*>0, i.e. a steady state. This basic observation is the content of corollary 2.4.

As the *x*_*i*_s represent probabilities,

1.1 $$x_{i}(t)\in [0,1]\;\text{for all}\;i\;\text{and}\;\sum_{j}x_{j}(t)=1,$$

for all *t*≥*t*_0_. The structure of the master equation guarantees that if *x*(*t*) satisfies (1.1) at time *t*=*t*_0_, then (1.1) holds for all *t*≥*t*_0_ even when the *x*_*i*_s are not necessarily linked to probabilities (see (1.9) below). Our results also hold of course in this case. The next example demonstrates such a case.

**Example 1.2**

An important topic in sociodynamics is the formation of large cities due to population migration. Haag [2], ch. 8 considers a master equation describing the flow of individuals between *N* settlements. The transition rates *p*_*ij*_ in this model represent the probability per time unit that an individual living in settlement *i* will migrate to settlement *j*. A mean-field approximation of this master equation yields a model in the form (1.2), where *x*_*i*_ represents the average density at settlement *i*, and $p_{ij}=\exp((x_{j}-x_{i})k_{ij})$, with *k*_*ij*_>0. This models the fact that the rate of transition from settlement *i* to settlement *j* increases when the population in settlement *j* is larger than in *i*, i.e. the tendency of individuals to migrate to larger cities. Note that the rates here are state-dependent, but not time-dependent. However, it is natural to assume that migration decisions depend on the season. For example, the tendency to migrate to colder cities may decrease (increase) in the winter (summer). This can be modelled by adding time dependence, say, changing the scaling parameters *k*_*ij*_ to functions *k*_*ij*_(*t*) that are periodic with a period of 1 year. Then the transition rates depend on both state and time, and are periodic.

It is important to note that, in general, nonlinear dynamical systems do not entrain to periodic excitations. Indeed, Nikolaev *et al.* [14] discusses two ‘simple looking’ nonlinear dynamical systems whose response to periodic forcing is chaotic (rather than periodic). Moreover, these systems commonly appear as components of larger sensing and signal transduction pathways in systems biology. This highlights the importance of proving that entrainment does hold in specific classes of dynamical systems.

Although entrainment has attracted enormous research attention, it seems that it has not been addressed before for the general case of systems modelled using a *T*-periodic master equation. Here we apply the theory of cooperative dynamical systems admitting a first integral to derive conditions guaranteeing that the answer to problem 1.1 is affirmative. In §3, we describe two applications of our approach to important systems from statistical physics. The first is the totally asymmetric simple exclusion process (TASEP). This model has been introduced in the context of biocellular processes [15] and has become the standard model for the flow of ribosomes along the mRNA molecule during translation [16,17]. More generally, TASEP has become a paradigmatic model for the statistical mechanics of non-equilibrium systems [6,7,18]. It is in particular used to study the stochastic dynamics of interacting particle systems such as vehicular traffic [19].

The second application is to an important model from epidemiology called the stochastic susceptible–infected–susceptible (SIS) model.

The remainder of this paper is organized as follows. In the following subsection, we briefly explain the central mathematical concepts used in the proofs of our main results theorems 2.2 and 2.5. The exact mathematical formulation of these results is provided in §2. The subsequent section then describes the two applications to statistical physics and to epidemiology mentioned above. This is followed by a brief discussion of the significance of the results and an outlook on possible future directions of research in §4. The appendix includes all the proofs. These are based on known tools, yet we are able to use the special structure of the master equation to derive stronger results than those available in the literature on monotone dynamical systems.

### 1.1. Formulation of master equation and concepts of proof

We begin by formulating the master equation, determined by given transition rates *p*_*ij*_(*t*,*x*)≥0, that governs the evolution of the probability distribution on *N* configurations:

1.2 $$\begin{array}{rl} {\dot{x}}_{1}(t) & =\sum_{\begin{array}{c} j=1\\ j\neq 1 \end{array}}^{N}p_{j1}(t,x(t))x_{j}(t)-\sum_{\begin{array}{c} j=1\\ j\neq 1 \end{array}}^{N}p_{1j}(t,x(t))x_{1}(t),\\ & \vdots \\ {\dot{x}}_{N}(t) & =\sum_{\begin{array}{c} j=1\\ j\neq N \end{array}}^{N}p_{jN}(t,x(t))x_{j}(t)-\sum_{\begin{array}{c} j=1\\ j\neq N \end{array}}^{N}p_{Nj}(t,x(t))x_{N}(t). \end{array}\}$$

In lemma A.4 below, it is shown in a slightly more general setting that system (1.2) defines a flow in the set of probability distributions $\left\{x\in {\left[0,1\right]}^{N}|\sum_{i=1}^{N}x_{i}=1\right\}$. For *T*>0, we refer to (1.2) as the *T*-periodic master equation if

1.3 $$p_{ij}(t+T,x)=p_{ij}(t,x),$$

for all *i*,*j*, all *t* and all *x*. Note that this includes the case where one (or several) of the rates is (are) *T*-periodic with *T*>0, and the other rates are time-independent, as a time-independent function satisfies (1.3) for all *T*. Clearly, from (1.3), it also follows that *p*_*ij*_(*t*+*kT*,*x*)=*p*_*ij*_(*t*,*x*) for any integer *k*. To make the *period* a well-defined notion one, therefore, often requires that the period is the *minimal* real number *T*>0 for which (1.3) is satisfied. Then constant functions do not have a period. As we want to include here the case of time-independent transition rates, e.g. corollary 2.3, we do not require the minimality of the common period *T* in (1.3).

For small values of *N*, it is sometimes possible to solve the master equation and then analyse entrainment directly. The next example demonstrates this.

**Example 1.3**

Consider the master equation (1.2) with *N*=2 and continuous time- (but not state-) dependent rates, i.e. *p*_*ij*_=*p*_*ij*_(*t*)≥0. Then (1.2) can be written as

1.4 $$\left[\begin{array}{c} {\dot{x}}_{1}\\ {\dot{x}}_{2} \end{array}\right]=\left[\begin{array}{cc} -p_{12} & p_{21}\\ p_{12} & -p_{21} \end{array}\right]\left[\begin{array}{c} x_{1}\\ x_{2} \end{array}\right].$$

Assume also that all the rates are periodic with period *T*>0. Using the fact that *x*_1_(*t*)+*x*_2_(*t*)≡1 yields

1.5 $${\dot{x}}_{1}(t)=p_{21}(t)-(p_{12}(t)+p_{21}(t))x_{1}(t).$$

Recall that *x*_1_(0),*x*_2_(0)∈[0,1] with *x*_1_(0)+*x*_2_(0)=1. Equation (1.5) implies that *x*_1_(*t*)∈[0,1], for all *t*≥0, and thus *x*_2_(*t*)∈[0,1], for all *t*≥0. Solving (1.5) yields

1.6 $$\begin{array}{rl} x_{1}(t) & =\exp\left(-\int_{0}^{t}(p_{12}(s)+p_{21}(s))\;ds\right)(x_{1}(0)+c(t))\\ \;\text{and}\;x_{2}(t) & =1-x_{1}(t), \end{array}\}$$

where $c(t):=\int_{0}^{t}p_{21}(\tau )\exp(\int_{0}^{\tau }(p_{12}(s)+p_{21}(s))\;ds)\;d\tau$. To analyse if every solution converges to a periodic solution, we consider two cases.

*Case* 1: If *p*_12_(*t*)+*p*_21_(*t*)≡0, then (1.6) yields *x*(*t*)≡*x*(0), i.e. every point in the state-space is an equilibrium point. This means in particular that every solution is a periodic solution with period *T*.

*Case* 2: Assume that there exists a time t*∈[0,*T*) such that *p*_12_(t*)+*p*_21_(t*)>0. (Note that by continuity this in fact holds on a time interval that includes t*.) The solution (1.6) is periodic with period *T* if and only if *x*_1_(*T*)=*x*_1_(0), i.e. if and only if

1.7 $$x_{1}(0)=\frac{\exp(-\int_{0}^{T}(p_{12}(s)+p_{21}(s))\;ds)c(T)}{1-\exp(-\int_{0}^{T}(p_{12}(s)+p_{21}(s))\;ds)}.$$

It is straightforward to show that the right-hand side in this equation is in [0,1], so in this case, there exists a unique periodic trajectory *γ*(*t*), with *γ*_1_(0) equal to the expression in (1.7) and *γ*_2_(0)=1−*γ*_1_(0). To determine if every trajectory converges to *γ*, let *z*(*t*):=*x*(*t*)−*γ*(*t*), that is, the difference between the solution emanating from *x*_0_ and the unique periodic solution. Then

$${\dot{z}}_{1}=-(p_{12}+p_{21})z_{1}\;\mathrm{and}\;z_{1}(0)=x_{1}(0)-\gamma_{1}(0).$$

As *p*_12_(*t*)+*p*_21_(*t*) is non-negative for all *t*, positive on a time interval and *T*-periodic, *z*_1_(*t*) converges to zero and we conclude that any trajectory of the system converges to the unique periodic solution *γ*.

Of course, when *N*>2 and the rates depend on both *t* and *x*, this type of explicit analysis is impossible, and the proof of entrainment requires a different approach.

In general, proving that a time-varying nonlinear dynamical system entrains to periodic excitations is non-trivial. Rigorous proofs are known for two classes of dynamical systems: contractive systems and monotone systems with additional structure like a tridiagonal Jacobian [20] or admitting a first integral.

A system is called *contractive* if any two trajectories approach one another at an exponential rate [21,22]. Such systems entrain to periodic excitations [9,23]. An important special case is asymptotically stable linear systems with an additive periodic input *u*, that is, systems in the form

1.8 $$\dot{x}=Ax+Bu,$$

with $x\in R^{N}$, $A\in R^{N\times N}$ a Hurwitz matrix,^1^ $u\in R^{M}$ and $B\in R^{N\times M}$. In this case, *x*(*t*) converges to a periodic solution *γ*(*t*) and it is also possible to obtain a closed-form description of *γ* using the transfer function of the linear system [24]. We note that even in the case that the *p*_*ij*_s in (1.2) do not depend on *x*, i.e. when (1.2) is linear in *x*, the master equation is not of the form (1.8) because the periodic influence in (1.2) enters through the transition rates *p*_*ij*_ and not through an additive input channel.

Next, we turn to the notion of a *first integral*. Define $H:R^{N}\to R$ by *H*(*y*):=*y*_1_+⋯+*y*_*N*_. Equation (1.2) implies that

1.9 $$\sum_{i=1}^{N}{\dot{x}}_{i}(t)\equiv 0,$$

so that the value of *H*(*x*(*t*)) remains constant under the flow, that is, *H* is a first integral of (1.2).

A system is called *monotone* if its flow preserves a partial order, induced by an appropriate cone *K*, between its initial conditions [25]. An important special case of monotone systems is cooperative systems for which the cone *K* is the positive orthant. To explain this, define a partial ordering between vectors $a,b\in R^{n}$ by *a*≤*b* if every entry of *a* is smaller or equal to the corresponding entry of *b*. For example, for vectors in $R^{3}$

[1.13.1211]≤[1.2411],

but

[1.13.1211]≰[1.2011].

A system $\dot{x}=f(x)$ is called cooperative if for any two initial conditions *a*,*b* with *a*≤*b* the solutions satisfy *x*(*t*,*a*)≤*x*(*t*,*b*) for any time *t*≥0. In other words, the dynamics preserves the ordering between the initial conditions.

Cooperative systems that admit a first integral entrain to periodic excitations. It is interesting to note that proofs of this property often follow from contraction arguments [26].

The master equation (1.2) is, in general, not contractive, although as we will show in theorem A.8 below it is on the ‘verge of contraction’ with respect to the ℓ_1_ vector norm (see [27] for some related considerations). However, (1.2) admits a first integral and is often a cooperative system (see theorem A.9 below). In particular, when the rates do not depend on the state, i.e. *p*_*ij*_=*p*_*ij*_(*t*), then (1.2) is always cooperative.

## 2. Main results

We begin by specifying the exact conditions on (1.2) that are assumed throughout. For a set *S*, let *int*(*S*) denote the interior of *S*. For any time *t*, *x*(*t*) is an *N*-dimensional column vector that includes the probabilities of all *N* possible configurations. The relevant state-space is thus

$$\Omega :=\left\{y\in R^{N}\;|\;y_{i}\geq 0\;\text{for all}\;i,\;\text{and}\;\sum_{i=1}^{N}y_{i}=1\right\}.$$

For an initial time *t*_0_≥0 and an initial condition *x*(*t*_0_), let *x*(*t*;*t*_0_,*x*(*t*_0_)) denote the solution of (1.2) at time *t*≥*t*_0_. For our purposes, it will be convenient to assume that the vector field associated with system (1.2) is not only defined on the set *Ω*, but on all the closed positive cones

$$R_{+}^{N}:=\{x\in R^{N}\;|\;x_{j}\geq 0\;\text{for all}\;1\leq j\leq N\}.$$

Throughout this paper, we assume that the following condition holds.

**Assumption 2.1**

There exists *T*>0 such that the transition rates *p*_*ij*_(*t*,*x*) are: continuous and non-negative on $[0,T]\times R_{+}^{N}$; continuously differentiable with respect to *x* on $\left[0,T)\times \mathrm{int}(R_{+}^{N}\right)$ and the derivative admits a continuous extension onto $[0,T)\times R_{+}^{N}$; and are jointly periodic with period *T*, that is,

2.1 $$p_{ij}(t+T,x)=p_{ij}(t,x),$$

for all *i*,*j*, all t∈[0,∞) and all $x\in R_{+}^{N}$.

Let relint(*Ω*) denote the relative interior of *Ω*, that is,

$$\mathrm{relint}(\Omega )=\left\{y\in R^{N}\;|\;y_{i}>0\;\text{for all}\;i,\;\text{and}\;\sum_{i=1}^{N}y_{i}=1\right\}.$$

Note that if the rates are only defined on *x*∈*Ω*, with partial derivatives with respect to *x*_*j*_ on relint(*Ω*) with continuous extensions to *Ω*, then they can be extended to $R_{+}^{N}$ so that the conditions in assumption 2.1 hold. For example, by defining them to be constant on rays through the origin and multiplied by a cut-off function *χ*(|*x*|_1_), where *χ* is a smooth function with a compact support in [0,∞), satisfying *χ*(*s*)=1 for *s*=1, and where |*x*|_1_ denotes the ℓ_1_-norm of *x*.

We now determine the conditions guaranteeing that (1.2) is a cooperative dynamical system. Note that (1.2) can be written as

2.2 $$\dot{x}(t)=f(t,x):=A(t,x(t))x(t),$$

where $A\in R^{N\times N}$ is a matrix with entries

2.3 $$a_{ij}(t,x(t)):=\left\{ \begin{array}{ll} p_{ji}(t,x(t)), & \text{if}\;i\neq j,\\ -\sum_{\begin{array}{c} k=1\\ k\neq i \end{array}}^{N}p_{ik}(t,x(t)), & \text{if}\;i=j. \end{array}\right.$$

The Jacobian of the vector field *f* is the *N*×*N* matrix

2.4 $$J(t,x):=\frac{\partial f(t,x)}{\partial x}=A(t,x)+B(t,x),$$

where *B* is the matrix with entries $b_{ij}:=\sum_{k=1}^{N}x_{k}(\partial a_{ik}/\partial x_{j})$. Recall that a matrix $M\in R^{n\times n}$ is called *Metzler* if every off-diagonal entry of *M* is non-negative. It follows that if $p_{ji}(t,x)+\sum_{k=1}^{N}x_{k}(\partial a_{ik}(t,x)/\partial x_{j})\geq 0$, for all *i*≠*j*, all *t*≥*t*_0_ and all *x*∈*Ω* then *J*(*t*,*x*) is Metzler for all *t*≥*t*_0_ and all *x*∈*Ω*.

We can now state our first result.

**Theorem 2.2**

*Suppose that
*

2.5 $$p_{ji}(t,x)+\sum_{k=1}^{N}\frac{\partial a_{ik}(t,x)}{\partial x_{j}}x_{k}\geq 0,\;\text{for all}\;i\neq j,\;t\geq t_{0},\;x\in \Omega .$$

*Then, for any t*_0_*≥0 and any x(t*_0_*)∈Ω the solution x(t;t*_0_*,x(t*_0_*)) of (*1.2*) converges to a periodic solution with period T.*

If the rates depend on time, but not on the state, i.e. *p*_*ij*_=*p*_*ij*_(*t*) for all *i*,*j*, then the condition in theorem 2.2 always holds, and this yields the following result.

**Corollary 2.3**

*If* *p*_*ij*_=*p*_*ij*_(*t*) *for all* *i*,*j*, *then for any* *t*_0_≥0 *and any* *x*(*t*_0_)∈*Ω* *the solution* *x*(*t*;*t*_0_,*x*(*t*_0_)) *of* (1.2) *converges to a periodic solution with period* *T*.

Thus, theorem 2.2 describes a technical condition guaranteeing entrainment, and this condition automatically holds in the case where all the rates are functions of time only.

If the rates depend on the state, but not on time then we may apply theorem 2.2 for all *T*>0. Thus, the trajectories converge to a periodic solution with an arbitrary period, i.e. a steady state. This yields the following result.

**Corollary 2.4**

*If* *p*_*ij*_=*p*_*ij*_(*x*) *for all* *i*,*j* *and in addition condition* (2.5) *holds, then for any* *t*_0_≥0 *and any* *x*(*t*_0_)∈*Ω* *the solution* *x*(*t*;*t*_0_,*x*(*t*_0_)) of (1.2) *converges to a steady state*.

In some applications, it is useful to establish that all trajectories of (1.2) converge to a *unique* periodic trajectory. Recall that a matrix $M\in R^{n\times n}$, with *n*≥2, is said to be *reducible* if there exists a permutation matrix *P*∈{0,1}^*n*×*n*^, and an integer 1≤*r*≤*n*−1 such that $P^{\prime }MP=\left[\begin{array}{cc} B & C\\ 0 & D \end{array}\right]$, where $B\in R^{r\times r}$, $D\in R^{\left(n-r)\times (n-r\right)}$, $C\in R^{r\times (n-r)}$ and $0\in R^{(n-r)\times r}$ is a zero matrix. A matrix is called *irreducible* if it is not reducible. It is well known that a Metzler matrix *M* is irreducible if and only if the graph associated with the adjacency matrix of *M* is strongly connected [28], theorems 6.2.14 and 6.2.24.

**Theorem 2.5**

*Suppose that the conditions in theorem* 2.2 hold and, furthermore, that there exists a time t*≥t_0_ such that A(t*,x)+B(t*,x) is an irreducible matrix for all x∈Ω. Then (1.2*) admits a unique periodic solution γ in Ω, with period T, and every solution x(t;t*_0_*,x(t*_0_*)) with x(t*_0_*)∈Ω converges to γ at an exponential rate.*

**Example 2.6**

Consider the system in example 1.3. This is of the form (2.2) with

$$A(t)=\left[\begin{array}{cc} -p_{12}(t) & p_{21}(t)\\ p_{12}(t) & -p_{21}(t) \end{array}\right].$$

If there exists a time t* such that *p*_12_(t*),*p*_21_(t*)>0 then *A*(t*) is irreducible. We conclude that, in this case, all the conditions in theorem 2.5 hold, so the system admits a unique *T*-periodic solution *γ* and every trajectory converges to *γ*. This agrees of course with the results of the analysis in example 1.3 above where we arrived at the same conclusion under the slightly weaker assumption that *p*_12_(t*)+*p*_21_(t*)>0.

The next section describes an application of our results to two important models.

## 3. Applications

### 3.1. Entrainment in totally asymmetric simple exclusion process

**Figure 1.**
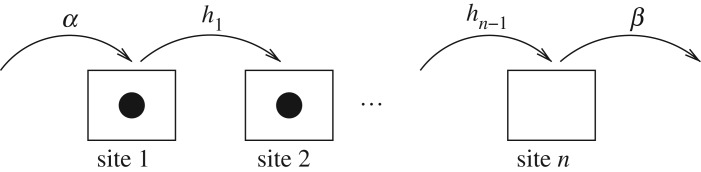
The TASEP model includes particles randomly hopping along a chain of *n* sites. Note that the particle in site 1 cannot hop forward because site 2 contains a particle.

The totally asymmetric simple exclusion process (TASEP) is a stochastic model of particles hopping along a one-dimensional chain. A particle at site *k* hops to site *k*+1 (the next site on the right) with an exponentially distributed probability^2^ with rate *h*_*k*_, provided the site *k*+1 is not occupied by another particle. This simple exclusion property generates an indirect link between the particles and allows to model the formation of traffic jams. Indeed, if a particle ‘gets stuck’ for a long time in the same site, then other particles accumulate behind it. At the left end of the chain particles enter with a certain entry rate *α*>0 and at the right end particles leave with a rate *β*>0 (figure 1).

As pointed out in the introduction, TASEP has become a standard tool for modelling ribosome flow during translation, and is a paradigmatic model for the statistical mechanics of non-equilibrium systems. We note that in the classical TASEP model the rates *α*, *β* and *h*_*i*_ are constants, but several papers considered TASEP with periodic rates [29–31] that can be used, for example, as models for vehicular traffic controlled by periodically varying traffic signals.

It was shown in [32] that the dynamic mean-field approximation of TASEP, called the ribosome flow model (RFM), entrains. However, the RFM is not a master equation and the proof of entrainment in [32] is based on different ideas. For more on the analysis of the RFM, see e.g. [33–36].

For a chain of length *n*, denoting an occupied site by 1 and a free site by 0, the set of possible configurations is {0,1}^*n*^, and thus the number of possible configurations is *N*=2^*n*^. The dynamics of TASEP can be expressed as a master equation with transition rates *p*_*ij*_ that depend on the values *α*, *β* and *h*_*i*_, *i*=1,…,*n*. For the sake of simplicity, we will show this in the specific case *n*=2, but all our results below hold for any value of *n*.

When *n*=2, the possible configurations of particles along the chain are *C*_1_:=(0,0), *C*_2_:=(0,1), *C*_3_:=(1,0) and *C*_4_:=(1,1). Let *x*_*i*_(*t*) denote the probability that the system is in configuration *C*_*i*_ at time *t*, for example, *x*_1_(*t*) is the probability that both sites are empty at time *t*. Then *x*_1_ may decrease (increase) due to the transition *C*_1_→*C*_3_ [*C*_2_→*C*_1_], i.e. when a particle enters the first site (a particle in the second site hops out of the chain). This gives

$${\dot{x}}_{1}(t)=-\alpha x_{1}(t)+\beta x_{2}(t).$$

Similar considerations for all configurations lead to the master equation $\dot{x}=Ax$, with

$$A:=\left(\begin{array}{cccc} -\alpha & \beta & 0 & 0\\ 0 & -\alpha -\beta & h_{1} & 0\\ \alpha & 0 & -h_{1} & \beta \\ 0 & \alpha & 0 & -\beta \end{array}\right).$$

If the entry, exit and hopping rates are time dependent and periodic, all with the same period *T*, one easily sees that the resulting master equation satisfies assumption 2.1 as well as all assumptions of theorem 2.2. Hence, we conclude that every solution of the master equation starting in *Ω* converges to a periodic solution with period *T*. Moreover, if there exists a time t* such that *α*(t*),*β*(t*),*h*_1_(t*)>0, then *A*(t*) is irreducible. Hence, the conditions of theorem 2.5 are also satisfied, so we conclude that the periodic solution is unique and convergence takes place at an exponential rate. It is not difficult to show that the same holds for TASEP with any length *n*.

**Example 3.1**

When *n*=3, the possible particle configurations are *C*_1_:= (0,0,0), *C*_2_:=(0,0,1), *C*_3_:=(0,1,0), *C*_4_:=(0,1,1),…,*C*_8_:=(1,1,1). Let *x*_*i*_(*t*) denote the probability that the system is in configuration *C*_*i*_ at time *t*. The TASEP master equation in this case is $\dot{x}=Ax$, with

$$A=\left[\begin{array}{cccccccc} -\alpha & \beta & 0 & 0 & 0 & 0 & 0 & 0\\ 0 & -\alpha -\beta & h_{2} & 0 & 0 & 0 & 0 & 0\\ 0 & 0 & -\alpha -h_{2} & \beta & h_{1} & 0 & 0 & 0\\ 0 & 0 & 0 & -\alpha -\beta & 0 & h_{1} & 0 & 0\\ \alpha & 0 & 0 & 0 & -h_{1} & \beta & 0 & 0\\ 0 & \alpha & 0 & 0 & 0 & -h_{1}-\beta & h_{2} & 0\\ 0 & 0 & \alpha & 0 & 0 & 0 & -h_{2} & \beta \\ 0 & 0 & 0 & \alpha & 0 & 0 & 0 & -\beta \end{array}\right].$$

**Figure 2.**
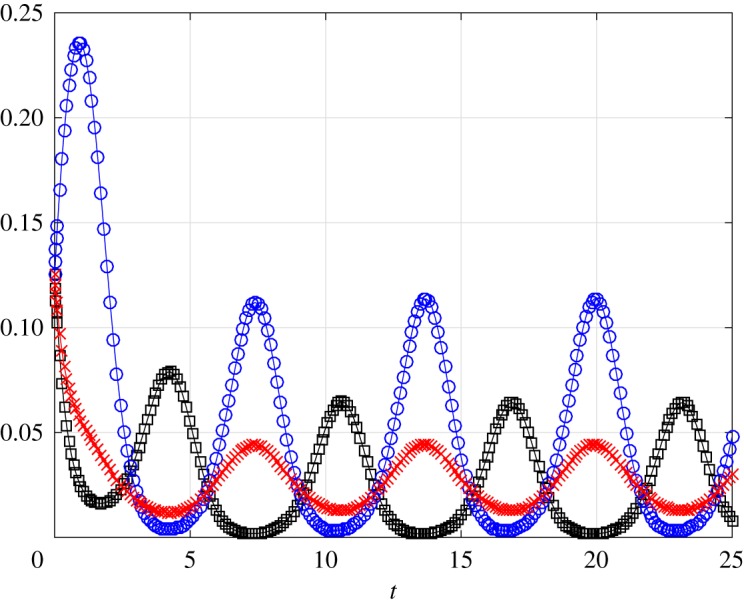
Probabilities *x*_1_(*t*) (black square), *x*_4_(*t*) (red asterisk) and *x*_8_(*t*) (blue circle) as a function of *t* in example 3.1.

We simulated this system with the rates

$$\alpha (t)=1+\cos(t),\;\beta (t)=1+\cos(t+\pi ),\;h_{1}=\frac{1}{2},\;h_{2}=\frac{1}{4}$$

and initial condition $x(0)={\left[\frac{1}{8}\;\ldots \;\frac{1}{8}\right]}^{\prime }$. Note that all the rates here are jointly periodic with period 2*π*. Figure 2 depicts *x*_1_(*t*) (black square), *x*_4_(*t*) (red asterisk) and *x*_8_(*t*) (blue circle) as a function of *t* (we depict only three *x*_*i*_s to avoid cluttering the figure). Note that as the entry rate *α*(*t*) is maximal and the exit rate *β*(*t*) is minimal at *t*=0, the probability *x*_8_(*t*) [*x*_1_(*t*)] to be in state (1,1,1) [(0,0,0)] quickly increases (decreases) near *t*=0. As time progresses, the probabilities converge to a periodic pattern with period 2*π*.

Entrainment of the probabilities *x*_*i*_ has consequences for other quantities of interest in statistical mechanics. For instance, an important quantity is the occupation density, i.e. the probability that site *k* is occupied, often denoted by 〈*τ*_*k*_〉, cf. [37,38]. Denoting the *k*th component of the configuration *C*_*i*_∈{0,1}^*n*^ by *C*_*i*,*k*_, a straightforward computation reveals that

$$\langle \tau_{k}(t)\rangle =\sum_{i=1}^{N}C_{i,k}x_{i}(t).$$

It is thus immediate that the occupation densities also converge to a unique periodic solution.

This phenomenon has already been observed empirically in [29] that studied a semi-infinite and finite TASEP coupled at the end to a reservoir with a periodic time-varying particle density. This models, for example, a traffic lane ending with a periodically varying traffic light. The simulations in [29] suggest that this leads to the development of a sawteeth density profile along the chain, and that ‘The sawteeth profile is changing with time, but it regains its shape after each complete period…’ [29], p. 011122-2 (see also [30,31] for some related considerations).

Our results can also be interpreted in terms of the particles along the chain in TASEP. As the expectation of the occupation densities 〈*τ*_*k*_〉 converges to a periodic solution, this means that, in the long term, the TASEP dynamics ‘fluctuates’ around a periodic ‘mean’ solution (see e.g. the simulation results depicted in fig. 5 in [32]). Moreover, in [30,31] it was found for closely related models that the limiting periodic density profiles (whose existence is also guaranteed by our results) have an interesting structure that depends in a non-trivial way on the frequency of the transition rates.

### 3.2 Entrainment in a stochastic susceptible–infected–susceptible model

The stochastic SIS model plays an important role in mathematical epidemiology [39]. But, as noted in [40], it is usually studied under the assumption of fixed contact and recovery rates. Here, we apply our results to prove entrainment in an SIS model with periodic rates.

**Figure 3.**
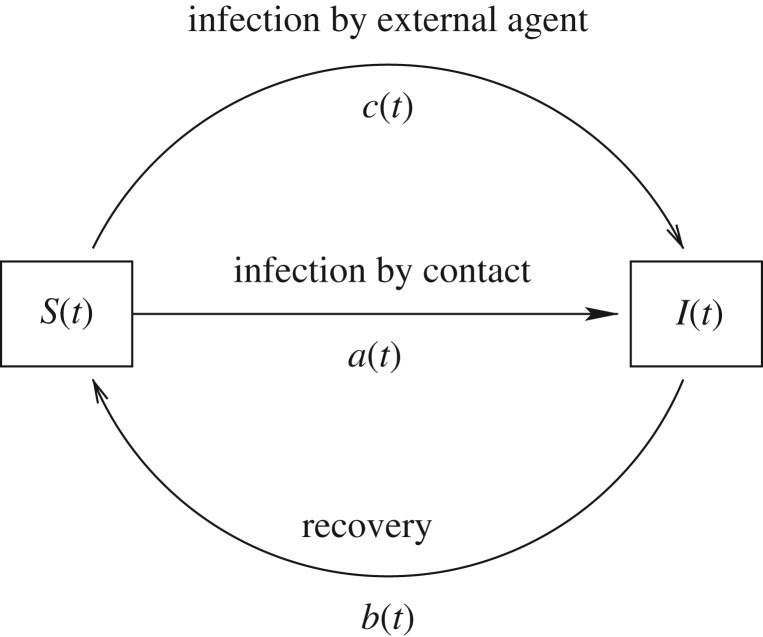
Three mechanisms for transitions between the classes of susceptible and infected and the associated rates.

Consider a population of size *N* divided into susceptible and infected individuals. Let *S*(*t*) [*I*(*t*)] denote the size of the susceptible (infected) part of the population at time *t*, so that *S*(*t*)+*I*(*t*)≡*N*. We assume two mechanisms for infection. The first is by contact with an infected individual and depends on the contact rate *a*(*t*). The second is by some external agent (modelling, say, insect bite) with rate *c*(*t*). The recovery rate is *b*(*t*) (figure 3). We assume that *a*(*t*), *b*(*t*) and *c*(*t*) are continuous and take non-negative values for all time *t*.

If *I*(*t*)=*n* (so *S*(*t*)=*N*−*n*), then the probability that one individual recovers in the time interval [*t*,*t*+*dt*] is *b*(*t*)*n* *dt*+*o*(*dt*), and the probability for one new infection to occur in this time interval is *a*(*t*)*n*((*N*−*n*)/*N*) *dt*+*c*(*t*)((*N*−*n*)/*N*) *dt*+*o*(*dt*). For *n*∈{0,…,*N*}, let *P*_*n*_(*t*) denote the probability that *I*(*t*)=*n*. This yields the master equation:

3.1 $${\dot{P}}_{n}=((n-1)a+c)\left(1-\frac{n-1}{N}\right)P_{n-1}-\left((na+c)\left(1-\frac{n}{N}\right)+nb\right)P_{n}+(n+1)bP_{n+1},$$

for *n*∈{0,1,…,*N*}, where we define *P*_−1_=*P*_*N*+1_:=0, and for simplicity omit the dependence on *t*. This set of *N*+1 equations may be written in matrix form as

$$\dot{x}=Mx,$$

where *x*:=[*P*_0_ *P*_1_ … *P*_*N*_]′∈[0,1]^*N*+1^, *M*:=*P*−*D*, with *D*:=diag(0,*b*,2*b*,…,*Nb*), and *P* is the (*N*+1)×(*N*+1) matrix:

$$\left[\begin{array}{cccccccc} -cq_{0} & b & 0 & 0 & \ldots & 0 & 0 & 0\\ cq_{0} & -(a+c)q_{1} & 2b & 0 & \ldots & 0 & 0 & 0\\ 0 & (a+c)q_{1} & -(2a+c)q_{2} & 3b & \ldots & 0 & 0 & 0\\ 0 & 0 & (2a+c)q_{2} & -(3a+c)q_{3} & \ldots & 0 & 0 & 0\\ & & & & \vdots & & & \\ 0 & 0 & 0 & 0 & \ldots & ((N-2)a+c))q_{N-2} & -((N-1)a+c)q_{N-1} & bN\\ 0 & 0 & 0 & 0 & \ldots & 0 & ((N-1)a+c)q_{N-1} & 0 \end{array}\right],$$

where *q*_*i*_:=1−*i*/*N*. Note that *M*(*t*) is Metzler, as *a*(*t*),*b*(*t*) and *c*(*t*) are non-negative for all *t*. Thus, theorems 2.2 and 2.5 yield the following result.

**Corollary 3.2**

*If* *a*(*t*), *b*(*t*) *and* *c*(*t*) *are all* *T*-*periodic then any solution of* (3.1) *with* $x(0)\in \Omega \subset R^{N+1}$ *converges to a* *T*-*periodic solution. Furthermore, if there exists a time* t*≥0 *such that*

3.2 $$b(t^{*})c(t^{*})>0,$$

*then there exists a unique* *T*-*periodic solution* *γ* *in* *Ω* *and every solution converges to* *γ*.

**Example 3.3**

**Figure 4.**
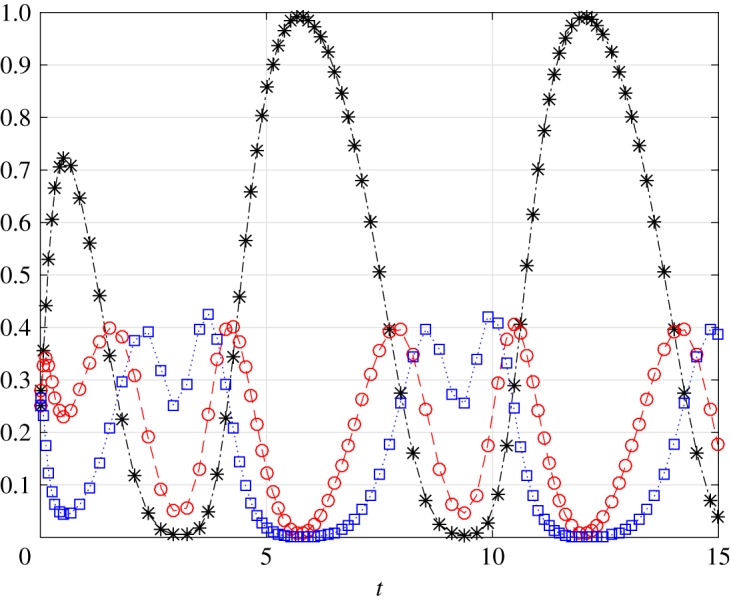
Probabilities *P*_0_(*t*) (black asterisk), *P*_1_(*t*) (red circle) and *P*_2_(*t*) (blue square) as a function of *t* in example 3.3.

Consider the stochastic SIS model with *N*=3, *a*(*t*)=1, b(t)=3+3cos⁡(t+0.5) and c(t)=2−2sin⁡(t+0.75). These rates are non-negative and jointly *T*-periodic for *T*=2*π* and clearly there exists t*≥0 such that *b*(t*)*c*(t*)>0. Figure 4 depicts *P*_*i*_(*t*), *i*=0,1,2, (note that *P*_3_(*t*)=1−*P*_0_(*t*)−*P*_1_(*t*)−*P*_2_(*t*)) as a function of time *t* for the initial condition $P(0)=(\frac{1}{4})1_{4}\in \Omega$. It may be seen that every *P*_*i*_(*t*) converges to a periodic solution with period 2*π*. Taking other initial conditions *x*(0)∈*Ω* yields convergence to the same periodic solution.

Note that if the irreducibility condition (3.2) does not hold then the system may have several periodic solutions. To see this, consider, for example, the case *b*(*t*)=*c*(*t*)≡0. Let $e^{i}\in R^{N+1}$ denote the vector with entry *i* equal to one and all other entries zero. Then both *x*(*t*)≡*e*^1^ and *x*(*t*)≡*e*^*N*+1^ are (periodic) solutions of the dynamics.

## 4. Discussion

In his 1929 paper on periodicity in disease prevalence, Soper [41] states: ‘Perhaps no events of human experience interest us so continuously, from generation to generation, as those which are, or seem to be, periodic.’ Soper also raised the question of whether the observed periodicity in epidemic outbreaks is the result of a ‘seasonal change in perturbing influences, such as might be brought about by school break-up and reassembling, or other annual recurrences?’ In modern terms, this amounts to asking whether the solutions of the system describing the dynamics of the epidemics entrain to periodic variations in the transmission parameters.

Here, we studied entrainment for dynamical systems described by a master equation. We considered a rather general formulation where the transition rates may depend on both time and state. Also, we did not assume any symmetry conditions (e.g. detailed balance conditions [3], ch. V) on the rates. We also note that this formulation implies similar results for nonlinear systems. Indeed, consider the time-varying nonlinear system:

4.1 $$\dot{x}=f(t,x)$$

and assume that *f*(*t*,0)=0, for all *t*. Let *J*(*t*,*x*):=(∂*f*/∂*x*)(*t*,*x*) denote the Jacobian of the vector field. Then

$$\begin{array}{rl} \dot{x} & =\int_{0}^{1}\frac{d}{ds}f(t,sx)\;ds\\ & =A(t,x)x, \end{array}$$

where $A(t,x):=\int_{0}^{1}J(t,sx)\;ds$. If *A*(*t*,*x*) has the form (2.3) then the results above can be applied to (4.1).

We proved that entrainment indeed holds under quite mild technical conditions. This follows from the fact that the master equation is a cooperative dynamical system admitting a first integral. Owing to the prevalence of the master equation as a model for natural and artificial phenomena, we believe that this result will find many applications. To demonstrate this, we described two applications of our results: a proof of entrainment in TASEP and in a stochastic SIS model.

The rigorous proof that the solutions of the master equation entrain is of course a necessary first step in studying the structure of the periodic trajectory (or trajectories), and its dependence on various parameters. Indeed, in many applications it is of interest to obtain more information on the periodic trajectory, e.g. its amplitude. Of course, one cannot expect in general to obtain a closed-form description of the limit cycle. However, for contractive dynamical systems there do exist efficient methods for obtaining a closed-form approximation of the limit cycle accompanied by explicit error bounds [23]. Developing a similar approach for the attractive limit cycle of the master equation may be an interesting topic for further research. In the specific case of TASEP with fixed rates, there exists a powerful representation of the steady state in terms of a product of matrices [7,37]. It may be of interest to try and represent the periodic steady state using a similar product, but with matrices with periodic entries. This could be used in particular to study the effects of periodic perturbations to the boundary-induced phase transitions that have been observed for TASEP in [42].

## Footnotes

1 That is, the real part of every eigenvalue of *A* is negative.

2 We consider the continuous time version of TASEP here.

## Data accessibility

All the relevant data are contained in this paper.

## Appendix A. Proofs of theorems 2.2 and 2.5

The proofs of theorems 2.2 and 2.5 are based on known tools from the theory of monotone dynamical systems admitting a first integral with a positive gradient (e.g. [43–45]). We present in this appendix a self-contained proof taking full advantage of the technical simplifications that our specific setting permits. This, in particular, allows us to prove that the results hold on the *closed* state-space and also that irreducibility at a single time point is enough to guarantee convergence to a unique periodic solution. Without loss of generality we always assume that the initial time is *t*_0_=0. It is convenient to work with the ℓ_1_ vector norm $|x{|}_{1}=\sum_{i}|x_{i}|$.

We begin by introducing some notation. First recall the notation

$$R_{+}^{N}:=\{x\in R^{N}\;|\;x_{j}\geq 0\;\text{for all}\;1\leq j\leq N\},$$

for the closed positive cone. Define a set of vector fields by

$$F:=\{f:[0,\infty )\times R_{+}^{N}\to R^{N}\;|\;\text{properties (i)–(v) below are satisfied}\},$$

where

(i) *f* is continuous;
(ii) for all *j*∈{1,…,*N*}, (∂*f*/∂*x*_*j*_)(*t*,*x*) exists for $\left(t,x)\in [0,\infty )\times \mathrm{int}(R_{+}^{N}\right)$ and has a continuous extension onto $[0,\infty )\times R_{+}^{N}$. Thus, *J*(*t*,*x*) from (2.4) is defined on $[0,\infty )\times R_{+}^{N}$ to be the continuous extension of (∂*f*/∂*x*_*j*_)(*t*,*x*);
(iii) *J*(*t*,*x*) is Metzler for all $(t,x)\in [0,\infty )\times R_{+}^{N}$;
(iv) $\sum_{i=1}^{N}f_{i}(t,x)=0$ for all $(t,x)\in [0,\infty )\times R_{+}^{N}$;
(v) *f*(*t*,0)=0 for all t∈[0,∞).

For *T*>0, let $F_{T}:=\{f\in F\;|\;f(t+T,x)=f(t,x)\;\text{for all}\;(t,x)\in [0,\infty )\times R_{+}^{N}\}$, that is, the set of vector fields in F that are also *T*-periodic.

It is straightforward to check that *f*(*t*,*x*)=*A*(*t*,*x*)*x* with *A* defined by (2.3) belongs to $F_{T}$ if assumption 2.1 and the assumptions of theorem 2.2 hold. Therefore, theorem 2.2 follows from the following result.

Theorem A.1 *If* $f\in F_{T}$ *then for all x*_0_*∈Ω the solution x(t;x*_0_*) of the initial value problem
*

$$\dot{x}=f(t,x),\;x(0)=x_{0},$$

*is asymptotically T-periodic, i.e. there exists a solution* γ:R→Ω *of* $\dot{\gamma }=f(t,\gamma )$*, with γ(t+T)=γ(t) for all* t∈R*, and
*

$$\lim_{t\to \infty }|x(t;x_{0})-\gamma (t){|}_{1}=0.$$

Let

$$F_{\mathrm{irr}}^{\Omega }:=\{f\in F\;|\;\text{there exists}\;t^{*}\geq 0\;\text{such that}\;J(t^{*},x)\;\text{is irreducible for all}\;x\in \Omega \}.$$

The next result is a generalization of theorem 2.5.

Theorem A.2 *If* $f\in (F_{T}\cap F_{\mathrm{irr}}^{\Omega })$ *then the differential equation* $\dot{x}=f(t,x)$ *admits a unique T-periodic solution* γ:R→Ω*. Moreover, there exists α>0 such that for any initial condition x*_0_*∈Ω the corresponding solution x(t;x*_0_*) satisfies
*

$$|x(t;x_{0})-\gamma (t){|}_{1}<2e^{-\alpha t}|x_{0}-\gamma (0){|}_{1},$$

*i.e. the solution converges to γ with exponential rate α.*

Complete proofs of theorems A.1 and A.2 are provided in the following seven subsections. We begin by showing in lemma A.4 that solutions of $\dot{x}=f(t,x)$, f∈F that start in the closed state-space $R_{+}^{N}$ are unique and remain in $R_{+}^{N}$ for all positive times. In the second subsection, we prove that for the subset of linear vector fields *f* in F the flow is cooperative, and non-expansive or even contractive in the case of irreducibility. The latter property is then generalized to the nonlinear setting (theorem A.8), which is enough to prove theorem A.2 in subsection A.4. The cooperative behaviour for nonlinear vector fields is stated in theorem A.9. In subsection A.6, we argue that the non-expansiveness of the flow together with the existence of a fixed point in the *ω*-limit set of the period map implies the asymptotic periodicity of the solution. The proof that such a fixed point exists is deferred to the final subsection. It uses the cooperative behaviour of the flow as well as the fact that the first integral *H* has a positive gradient, i.e. ∇*H*∈ int$\left(R_{+}^{N}\right)$.

**A.1. Positive invariance of $R_{+}^{N}$**

Our first goal is to establish in lemma A.4 below that for any $x_{0}\in R_{+}^{N}$ a unique solution *x*(*t*;*x*_0_) exists for all t∈[0,∞) and remains in the closed cone $R_{+}^{N}$. Denote

$$B:=\left\{B\in R^{N\times N}\;\left|\;B\;\text{is Metzler and}\;\sum_{i=1}^{N}B_{ij}=0\;\text{for all}\;j\in \{1,\ldots ,N\right\}\right\}.$$

Proposition A.3 *Assume that* f∈F. *Then there exists a continuous map* $B:[0,\infty )\times R_{+}^{N}\times R_{+}^{N}\to B$ *such that*

A 2 $$f(t,x)-f(t,y)=B(t,x,y)(x-y)\;\text{for all}\;t\geq 0\;\text{and all}\;x,y\in R_{+}^{N}.$$

*Moreover, for any index* *j* *the following property holds. If* $x\in R_{+}^{N}$ *with* *x*_*j*_=0 *then* *f*_*j*_(*t*,*x*)≥0 *for all* *t*≥0.

Proof. Equation (A 2) follows from the fundamental theorem of calculus with

$$B(t,x,y)=\int_{0}^{1}J(t,y+s(x-y))\;ds\;\text{for all}\;t\geq 0\;\text{and}\;x,y\in \mathrm{int}(R_{+}^{N}).$$

By assumptions (iii) and (iv) on *f*, we conclude that B(t,x,y)∈B. Moreover, assumption (ii) implies that this formula actually defines *B* as a continuous map on $[0,\infty )\times R_{+}^{N}\times R_{+}^{N}$ into B. Equation (A 2) also holds on the closed cone $R_{+}^{N}$ due to assumption (i). The second claim follows from *f*(*t*,*x*)=*B*(*t*,*x*,0)*x* and the fact that *B*(*t*,*x*,0) is Metzler. ▪

Lemma A.4 *Assume that* f∈F. *Then for every* $x_{0}\in R_{+}^{N}$ *the initial value problem* $\dot{x}=f(t,x)$, *x*(0)=*x*_0_, *admits a unique solution* $x(\;\cdot \;;x_{0}):[0,\infty )\to R_{+}^{N}$. *Moreover*,

$$H(x)=\sum_{j=1}^{N}x_{j}$$

*is a first integral of the dynamics, i.e*. (*d*/*dt*)*H*(*x*(*t*;*x*_0_))≡0.

Proof. By assumption (i) on *f* and by proposition A.3, the vector field *f* satisfies the hypotheses of the Picard–Lindelöf theorem, however, with a domain of definition $R_{+}^{n}$ which is not open. Introduce the auxiliary extension

$$\tilde{f}(t,x):=f(t,|x_{1}|,\ldots ,|x_{N}|).$$

Note that $\tilde{f}:[0,\infty )\times R^{N}\to R^{N}$ is well defined. The Picard–Lindelöf theorem yields existence and uniqueness of a maximal solution $\tilde{x}(\;\cdot \;;x_{0}):J_{x_{0}}\to R^{N}$ of the initial value problem $\dot{x}=\tilde{f}(t,x)$, *x*(0)=*x*_0_, with *J*_*x*_0__=[0,*b*_*x*_0__) for some *b*_*x*_0__>0 that might be infinite. Note that *H* is a first integral by property (iv) of *f*, that carries over to $\tilde{f}$. On $R_{+}^{N}$, the solutions *x*( ⋅ ;*x*_0_) and $\tilde{x}(\;\cdot \;;x_{0})$ coincide. We now show that for $x_{0}\in R_{+}^{N}$ the solution $\tilde{x}(t;x_{0})\in R_{+}^{N}$ for all *t*∈*J*_*x*_0__. If *x*_0_=0 then $\tilde{x}(t;x_{0})\equiv 0$ by assumption (v) on *f*, so $\tilde{x}(t;x_{0})\in R_{+}^{N}$. If $x_{0}\in R_{+}^{N}\setminus \{0\}$, we argue by contradiction. Assume that there exists *τ*>0 such that $\tilde{x}(\tau ;x_{0})\in (R^{N}\setminus R_{+}^{N})$. For ε∈R, let

$${\tilde{f}}_{\varepsilon }:[0,\infty )\times R^{N}\to R^{N},\;{\tilde{f}}_{\varepsilon }(t,x):=\tilde{f}(t,x)+\varepsilon \left(\begin{array}{ccc} 1 & \cdots & 1\\ \vdots & \vdots & \vdots \\ 1 & \cdots & 1 \end{array}\right)x-\varepsilon Nx$$

and denote by $\tilde{x}(\cdot ;x_{0},\varepsilon )$ the unique maximal solution of $\dot{x}={\tilde{f}}_{\varepsilon }(t,x)$, *x*(0)=*x*_0_. Note that by the definition of ${\tilde{f}}_{\varepsilon }$ the function *H* is also a first integral for this dynamical system. Now by the continuous dependence of solutions on parameters there exists *ε*_0_>0 such that $\tilde{x}(\tau ;x_{0},\varepsilon_{0})\in (R^{N}\setminus R_{+}^{N})$. This implies that there exists a time *s*∈[0,*τ*) such that *x*(*s*;*x*_0_,*ε*_0_) leaves $R_{+}^{N}$, i.e. there exists *k*∈{1,…,*N*} with

A 3 $${\tilde{x}}_{k}(s;x_{0},\varepsilon_{0})=0\;\text{and}\;{\dot{\tilde{x}}}_{k}(s;x_{0},\varepsilon_{0})\leq 0.$$

We will show that this is a contradiction. As ${\dot{\tilde{x}}}_{k}(s;x_{0},\varepsilon_{0})={\left({\tilde{f}}_{\varepsilon }(s,\tilde{x}(s;x_{0},\varepsilon_{0}))\right)}_{k}$, the definition of ${\tilde{f}}_{\varepsilon }$ and (A 3) yield ${\dot{\tilde{x}}}_{k}(s;x_{0},\varepsilon_{0})={\left(\tilde{f}(s,\tilde{x}(s;x_{0},\varepsilon_{0}))\right)}_{k}+\varepsilon_{0}\sum_{i=1}^{N}{\tilde{x}}_{i}(s;x_{0},\varepsilon_{0})$. By the second statement in proposition A.3, ${\left(\tilde{f}(s,\tilde{x}(s;x_{0},\varepsilon_{0}))\right)}_{k}\geq 0$, so ${\dot{\tilde{x}}}_{k}(s;x_{0},\varepsilon_{0})\geq \varepsilon_{0}\sum_{i=1}^{N}{\tilde{x}}_{i}(s;x_{0},\varepsilon_{0})$. As *H* is also a first integral for $\dot{y}={\tilde{f}}_{\varepsilon }(t,y)$, we obtain ${\dot{\tilde{x}}}_{k}(s;x_{0},\varepsilon_{0})\geq \varepsilon_{0}H(x_{0})>0$. This contradicts (A 3). We have established that $\tilde{x}$ remains in the compact set $\left\{z\in R_{+}^{N}\;|\;H(z)=H(x_{0})\right\}$ and it follows that the solution $\tilde{x}(t;x_{0})$ exists for all *t*≥0. As the solutions *x*( ⋅ ;*x*_0_) and $\tilde{x}(\;\cdot \;;x_{0})$ coincide on $R_{+}^{N}$, this completes the proof. ▪

**A.2. Linear time-varying systems**

The properties that are essential in the proofs of our main results are cooperativeness, non-expansiveness and contractivity of the flow. As it turns out, it is convenient to first prove these properties for linear time-varying systems. Let

A:={A:[0,∞)→B | A is continuous}.

Lemma A.5 *Assume that* A∈A. *Then the initial value problem* $\dot{x}=A(t)x$, $x(0)=x_{0}\in R_{+}^{N}$, *has a unique solution* $x(\;\cdot \;;x_{0}):[0,\infty )\to R^{N}$ *that satisfies the following properties*:

(a) $x(t;x_{0})\in R_{+}^{N}$ *and* *H*(*x*(*t*;*x*_0_))=*H*(*x*_0_) *for all* *t*≥0.
(b) *If* *x*_*j*_(*t**;*x*_0_)>0 *for some* *j*∈{1,…,*N*} *and* *t**≥0, *then* *x*_*j*_(*t*;*x*_0_)>0 *for all* *t*≥*t**.
(c) *If* *x*_0_≠0 *and* *A*(*t**) *is irreducible for some* *t**≥0, *then* $x(t;x_{0})\in \mathrm{int}(R_{+}^{N})$ *for all* *t*>*t**.

Proof. Existence and uniqueness of the solution are immediate from the linearity and continuity of *A*. The proof of (a) follows from lemma A.4 and the fact that *f*(*t*,*x*):=*A*(*t*)*x* belongs to F for each A∈A. To prove (b), assume that *x*_*j*_(*t**;*x*_0_)>0. Let *y*(*t*):=*x*_*j*_(*t*;*x*_0_). Then *y* solves the scalar initial value problem

$$\dot{y}=g(t,y),\;y(t^{*})=x_{j}(t^{*};x_{0})>0,$$

where

$$g(t,y):=a_{jj}(t)y+b(t),\;b(t):=\sum_{k\neq j}a_{jk}(t)x_{k}(t;x_{0})\geq 0.$$

Thus, letting $q(t):=\int_{t^{*}}^{t}a_{jj}(u)\;du$ yields

$$\begin{array}{rl} y(t) & =e^{q(t)}\left(y(t^{*})+\int_{t^{*}}^{t}e^{-q(s)}b(s)\;ds\right)\\ & \geq e^{q(t)}y(t^{*})\\ & >0, \end{array}$$

for all *t*≥*t**. To prove property (c), first note that irreducibility of *A*(*t**) implies that there exists *δ*>0 such that

A 4 $$A(t)\;\text{is irreducible for all}\;t\in [t^{*},t^{*}+\delta ).$$

This follows from the fact that irreducibility is equivalent to the associated adjacency graph being strongly connected, i.e. certain edges have positive weights, and the continuity of *A*(*t*). Pick $x_{0}\in R_{+}^{n}\setminus \{0\}$. We consider two cases. *Case* 1: $x(t^{*};x_{0})\in \mathrm{int}(R_{+}^{N})$. Then the claim follows from property (b). *Case* 2: $x(t^{*};x_{0})\in \partial R_{+}^{N}$. Fix *t*>*t**. As *H*(*x*(*t**;*x*_0_))=*H*(*x*_0_)>0, there exists *k*∈{1,…,*N*−1} such that exactly *k* entries of *x*(*t**;*x*_0_) are positive and the other entries are zero. Note that by property (b), these *k* entries remain positive for all *t*≥*t**. Assume w.l.o.g. that the first *k* entries of *x*(*t**;*x*_0_) are positive. Then in block form

$$\dot{x}(t^{*};x_{0})=\left(\begin{array}{cc} U & V\\ Y & Z \end{array}\right)\left(\begin{array}{c} x_{1}(t^{*};x_{0})\\ \vdots \\ x_{k}(t^{*};x_{0})\\ 0 \end{array}\right),$$

with $U\in R^{k\times k}$ and $Z\in R^{\left(N-k)\times (N-k\right)}$. As *A*(*t**) is Metzler and irreducible, every entry of *Y* is non-negative and at least one entry is positive, so there exists *j*>*k* such that ${\dot{x}}_{j}(t^{*};x_{0})>0$. Therefore, at least *k*+1 entries of *x*(*t*;*x*_0_) are positive for *t*>*t**. Now an inductive argument and using (A 4) completes the proof. ▪

Let

$$S:=\left\{Q\in R^{N\times N}\;|\;q_{jk}\geq 0\;\text{for all}\;j,k=1,\ldots ,N\;\text{and}\;\sum_{j=1}^{N}q_{jk}=1\;\text{for all}\;k=1,\ldots ,N\right\}$$

denote the set of *N*×*N* stochastic matrices, and let

$$S^{+}:=\{Q\in S\;|\;q_{jk}>0\;\text{for all}\;j,k=1,\ldots ,N\}$$

denote the subset of stochastic matrices with positive entries. For A∈A let $\Phi_{A}:[0,\infty )\to R^{N\times N}$ be the fundamental matrix of $\dot{x}=Ax$, that is, the solution of

$$\dot{\Phi }(t)=A(t)\Phi (t),\;\Phi (0)=I_{N}.$$

As the columns of *Φ*_*A*_(*t*) are *x*(*t*;*e*^*j*^), where $e^{j}\in R_{+}^{N}$ denotes the *j*th canonical unit vector, the next result follows from properties (a) and (c) in lemma A.5.

Corollary A.6 *Assume that* A∈A. *Then*

(a) $\Phi_{A}(t)\in S$ *for all* *t*≥0.
(b) *If* *A*(*t**) *is irreducible for some* *t**≥0, *then* $\Phi_{A}(t)\in S^{+}$ *for all* *t*>*t**.

We now use this to prove non-expansiveness with respect to the ℓ_1_-norm and contractivity in the case of irreducibility. The first step is to note that stochastic matrices have useful properties with respect to this norm.

Proposition A.7 *If* Q∈S *and* $x\in R^{N}$, *then* |*Qx*|_1_≤|*x*|_1_. *If* $Q\in S^{+}$ *and* $x\in (R^{n}\setminus \{0\})$ *with* *H*(*x*)=0, *then* |*Qx*|_1_<|*x*|_1_.

Proof. The first statement follows from

A 5 $$\begin{array}{rl} |Qx{|}_{1} & =\sum_{j=1}^{N}\left|\sum_{k=1}^{N}Q_{jk}x_{k}\right|\\ & \leq \sum_{j=1}^{N}\sum_{k=1}^{N}Q_{jk}|x_{k}|\\ & =\sum_{k=1}^{N}|x_{k}|\\ & =|x{|}_{1}. \end{array}$$

To prove the second statement, pick *x*≠0 such that *H*(*x*)=0. Then there exist *k*_1_,*k*_2_∈{1,…,*N*} such that *x*_*k*_1__<0<*x*_*k*_2__. Thus, if $Q\in S^{+}$ then for any *j*∈{1,…,*N*},

$$\left|\sum_{k=1}^{N}Q_{jk}x_{k}\right|<\sum_{k=1}^{N}Q_{jk}|x_{k}|$$

because the sum on the left contains both positive and negative terms. Now arguing as in (A 5) completes the proof. ▪

**A.3. Non-expansiveness and contractivity**

Using the results for time-varying linear systems, we now turn to proving non-expansiveness and contractivity for the nonlinear dynamical system.

Theorem A.8 *Suppose that* f∈F*. Then*

(a) *For any* $x_{0},y_{0}\in R_{+}^{N}$ *the function
  *
  A 6 $$t\mapsto |x(t;x_{0})-x(t;y_{0}){|}_{1}\;\text{is non-increasing on}\;[0,\infty ).$$
(b) *If there exists t***≥0 such that J(t***,x) is irreducible for all x∈Ω then for any* $\hat{t}>t^{*}$ *there exists* $M_{\hat{t}}<1$ *such that
  *
  A 7 $$|x(\hat{t};x_{0})-x(\hat{t};y_{0}){|}_{1}\leq M_{\hat{t}}|x_{0}-y_{0}{|}_{1}\;\text{for all}\;x_{0},y_{0}\in \Omega .$$

Proof. Fix *t*_1_≥0 and *x*_0_,*y*_0_∈*Ω*. Let *z*(*t*):=*x*(*t*+*t*_1_;*x*_0_)−*x*(*t*+*t*_1_;*y*_0_). By proposition A.3, $\dot{z}(t)=C(t)z(t)$, with *z*(0)= *z*_0_:=*x*(*t*_1_;*x*_0_)−*x*(*t*_1_;*y*_0_), and C∈A given by

$$C(t):=B(t+t_{1},x(t+t_{1};x_{0}),x(t+t_{1};y_{0})),$$

for all *t*≥0. Hence, *z*(*t*)=*Φ*_*C*_(*t*)*z*_0_ for all *t*≥0. To prove (A 6), pick *t*_2_≥*t*_1_. By corollary A.6(a), $\Phi_{C}(t_{2}-t_{1})\in S$ and proposition A.7 yields

$$\begin{array}{rl} |x(t_{2};x_{0})-x(t_{2};y_{0}){|}_{1} & =|z(t_{2}-t_{1}){|}_{1}\\ & =|\Phi_{C}(t_{2}-t_{1})z_{0}{|}_{1}\\ & \leq |z_{0}{|}_{1}\\ & =|x(t_{1};x_{0})-x(t_{1};y_{0}){|}_{1} \end{array}$$

and this proves (A 6). To prove (A 7), let

$$P:=\{x\in R^{N}\;|\;H(x)=0\;\text{and}\;|x{|}_{1}=1\}.$$

Note that if *y*,*z*∈*Ω* with *y*≠*z* then $(y-z)/|y-z{|}_{1}\in P$. Pick $\hat{t}>t^{*}$ and *x*_0_,*y*_0_∈*Ω*. Equation (A 7) clearly holds if *x*_0_=*y*_0_, so we may assume that *x*_0_≠*y*_0_. Then

$$\begin{array}{rl} |x(\hat{t};x_{0})-x(\hat{t};y_{0}){|}_{1} & =|\Phi_{C}(\hat{t})(x_{0}-y_{0}){|}_{1}\\ & =m\left(x_{0},y_{0},\frac{x_{0}-y_{0}}{|x_{0}-y_{0}{|}_{1}}\right)|x_{0}-y_{0}{|}_{1}, \end{array}$$

where m:Ω×Ω×P→R is defined by $m(x_{0},y_{0},v):=|\Phi_{C}(\hat{t})v{|}_{1}$. Let

$$M_{\hat{t}}:=\max\{m(x,y,v)\;|\;x\in \Omega ,y\in \Omega ,v\in P\}.$$

This is well defined, as the matrix *C*(*t*)=*C*_*x*_0_,*y*_0__(*t*)=*B*(*t*,*x*(*t*;*x*_0_),*x*(*t*;*y*_0_)) depends continuously on *x*_0_,*y*_0_∈*Ω* for all *t*≥0 (see proposition A.3) and thus $\Phi_{C_{x_{0},y_{0}}}(\hat{t})$ is also continuous in *x*_0_,*y*_0_. We conclude that

$$|x(\hat{t};x_{0})-x(\hat{t};y_{0}){|}_{1}\leq M_{\hat{t}}|x_{0}-y_{0}{|}_{1}.$$

Thus, to complete the proof we only need to show that $M_{\hat{t}}<1$. To prove this, denote *x**:=*x*(*t**;*x*_0_), *y**:=*x*(*t**;*y*_0_). Then

$$C_{x_{0},y_{0}}(t^{*})=B(t^{*},x^{*},y^{*})=\int_{0}^{1}J(t^{*},y^{*}+s(x^{*}-y^{*}))\;ds.$$

As *J* is Metzler (i.e. all its off-diagonal elements are non-negative) and, by assumption, *J*(*t**,*z*) is irreducible for all *z*∈*Ω*, we conclude that *C*_*x*_0_,*y*_0__(*t**) is also irreducible. Corollary A.6 implies that $\Phi_{C_{x_{0},y_{0}}}(\hat{t})\in S^{+}$. Picking a maximizer $(x_{0},y_{0},v_{0})\in \Omega \times \Omega \times P$ of *m*, i.e. $M_{\hat{t}}=m(x_{0},y_{0},v_{0})$, it follows from proposition A.7 that $M_{\hat{t}}<|v_{0}{|}_{1}=1$. ▪

**A.4. Proof of theorem A.2**

We can now prove theorem A.2. We note that the proof proceeds without the explicit use of the cooperative behaviour of dynamical systems (though we will use this property for the proof of theorem A.1; see subsections A.5 and A.6).

Note that for $f\in F_{T}$ a solution $\dot{\gamma }=f(t,\gamma )$, γ:[0,∞)→Ω, is *T*-periodic if and only if *x*(*T*;*γ*(0))=*γ*(0). Thus, consider the period map $P_{T}:R_{+}^{N}\to R_{+}^{N}$ defined by

A 8 $$P_{T}(a):=x(T;a).$$

In other words, *P*_*T*_(*a*) is the value of *x*(*T*) for the initial condition *x*(0)=*a*. Observe that *P*_*T*_(*Ω*)⊆*Ω* (as *H* is a first integral of $\dot{x}=f(t,x)$). Moreover, for $f\in (F_{T}\cap F_{\mathrm{irr}}^{\Omega })$ there exists *t**∈[0,*T*) such that *J*(*t**,*x*) is irreducible for all *x*∈*Ω*. Then *T*>*t**, so theorem A.8(b) implies that *P*_*T*_ is Lipschitz on the closed set *Ω* with Lipschitz constant *M*_*T*_<1. The Banach fixed point theorem implies that *P*_*T*_ has a unique fixed point in *Ω*, that is, there exists a unique *T*-periodic function γ:R→Ω that solves $\dot{\gamma }=f(t,\gamma )$. Fix *α*>0 such that

A 9 $$\max\{\frac{1}{2},M_{T}\}\leq e^{-\alpha T}.$$

Pick *x*_0_∈*Ω* and *t*≥0, and let $k\in N_{0}$ be such that *kT*≤*t*≤(*k*+1)*T*. Then theorem A.8 yields

$$\begin{array}{rl} |x(t;x_{0})-\gamma (t){|}_{1} & \leq |x(kT;x_{0})-\gamma (kT){|}_{1}\\ & \leq {\left(M_{T}\right)}^{k}|x_{0}-\gamma (0){|}_{1}\\ & \leq e^{-\alpha Tk}|x_{0}-\gamma (0){|}_{1}\\ & =e^{-\alpha T(k+1)}e^{\alpha T}|x_{0}-\gamma (0){|}_{1}\\ & \leq e^{\alpha T}e^{-\alpha t}|x_{0}-\gamma (0){|}_{1}\\ & \leq 2e^{-\alpha t}|x_{0}-\gamma (0){|}_{1}, \end{array}$$

where the last inequality follows from (A 9). Thus, every solution *x*(*t*;*x*_0_) converges to the unique periodic solution *γ*(*t*) at an exponential rate.

**A.5. Cooperative behaviour**

For the proof of theorem A.1, we use some elegant topological ideas from [44,45], which are based on the cooperative behavior of the dynamical system generated by a vector field f∈F. To formulate this concept, we introduce some more notation.

Let $p,q\in R^{N}$, and let *A* be a bounded non-empty subset of $R^{N}$. Then we write

(a) *p*≤*q* :⇔ *p*_*j*_≤*q*_*j*_ for all *j*∈{1,…,*N*},
(b) $\left[p,q]:=\{x\in R^{N}\;|\;p\leq x\leq q\right\}$,
(c) $\inf A:=c\in R^{N}$ with $c_{j}:=\inf\{a_{j}\;|\;a\in A\}$, $\sup A:=d\in R^{N}$
  with $d_{j}:=\sup\{a_{j}\;|\;a\in A\}$,
(d) *p*≤*A* ⇔ *p*≤*a* for all *a*∈*A*
  *q*≥*A* ⇔ *q*≥*a* for all *a*∈*A*.

It is straightforward to verify that infA≤A≤supA and that for all $x,y\in R^{N}$ with *x*≤*A*, *y*≥*A* we have x≤infA and y≥supA.

The following theorem summarizes the monotone behaviour with respect to the order ≤.

Theorem A.9 *Let* f∈F *and* $x_{0},y_{0}\in R_{+}^{N}$ *with x*_0_*≤y*_0_*. Then x(t;x*_0_*)≤x(t;y*_0_*) for all t≥0. If, in addition, (x*_0_)_*j*_*<(y*_0_)_*j*_ *for some j∈{1,…,N}, then x*_*j*_(*t;x*_0_)<*x*_*j*_(*t;y*_0_) *for all t≥0.*

Proof. We use again that *z*(*t*):=*x*(*t*;*y*_0_)−*x*(*t*;*x*_0_) satisfies $\dot{z}=C(t)z$, $z(0)=z_{0}:=y_{0}-x_{0}\in R_{+}^{N}$ with $C(t):=B(t,x(t;y_{0}),x(t;x_{0}))\in B$ and C∈A. The claim then follows from statements (a) and (b) of lemma A.5. ▪

**A.6. *ω*-Limit sets of *P*_*T*_ and proof of theorem A.1**

The concept of *ω*-limit sets for the discrete time dynamical system induced by the period map *P*_*T*_:*Ω*→*Ω* from (A 8) is pivotal for the proof of theorem A.1. For *a*∈*Ω* this set is

$$\omega_{T}(a):=\{x\in \Omega \;|\;\text{there is a sequence}\;n_{k}\to \infty \;\text{with}\;\lim_{k\to \infty }P_{T}^{n_{k}}(a)=x\}.$$

We first state a few standard facts about this set.

Proposition A.10 *Let* f∈F. *Pick* *T*>0. *Then for all* *a*∈*Ω* *we have*

(a) *ω*_*T*_(*a*) *is a closed, non-empty subset of* *Ω*;
(b) *P*_*T*_(*ω*_*T*_(*a*))=*ω*_*T*_(*a*);
(c) *ω*_*T*_(*b*)⊆*ω*_*T*_(*a*) *for all* *b*∈*ω*_*T*_(*a*).

Proof. Statements (a) and (b) follow from [46], eqn. (4.7.2) and lemma 4.7.4 because $P_{T}^{k}(a)$ evolves in the compact set *Ω*. To prove (c), pick *b*∈*ω*_*T*_(*a*). Property (b) implies that $P_{T}^{n_{k}}(b)\in \omega_{T}(a)$ for all $n_{k}\in N$, and as *ω*_*T*_(*a*) is closed $\lim_{k\to \infty }P_{T}^{n_{k}}(b)\in \omega_{T}(a)$. ▪

The following lemma, that is proved in the subsequent subsection, provides all that is needed to prove theorem A.1.

Lemma A.11 *Let* $f\in F_{T}$. *Then for every* *x*∈*Ω* *its limit set* *ω*_*T*_(*x*) *contains a fixed point of* *P*_*T*_.

Proof of theorem A.2 Pick *x*_0_∈*Ω* and denote by *z*∈*ω*_*T*_(*x*_0_) the fixed point of *P*_*T*_ that exists according to lemma A.11. As *z* is a fixed point and as $f\in F_{T}$ is *T*-periodic, the solution *γ*(*t*):=*x*(*t*;*z*) is also *T*-periodic. As *z*∈*ω*_*T*_(*x*_0_) there exists a subsequence $P_{T}^{n_{k}}(x_{0})\to z$ as k→∞. For any j∈N and *t*≥*n*_*j*_*T*, we have by theorem A.8(a),

$$|x(t;x_{0})-\gamma (t){|}_{1}\leq |x(Tn_{j};x_{0})-\gamma (Tn_{j}){|}_{1}=|P_{T}^{n_{j}}(x_{0})-z{|}_{1}.$$

As t→∞, we can take j→∞ and this yields

$$\lim_{t\to \infty }|x(t;x_{0})-\gamma (t){|}_{1}=0,$$

proving theorem A.1. ▪

Note that the proof of theorem A.1 shows that for any *x*_0_∈*Ω* the *ω*-limit set *ω*_*T*_(*x*_0_) cannot contain more than one fixed point of *P*_*T*_. Thus, the statement in lemma A.11 can actually be strengthened to *ω*_*T*_(*x*_0_) contains exactly one fixed point of *P*_*T*_.

The next subsection contains the proof of the crucial lemma A.11. We have adapted the ideas presented by Ji-Fa in [45] to our setting, which led to somewhat simplified arguments. In particular, we can replace [44], proposition 1 that is used in the proof of lemma 3.2 of [45] by a standard application of Brouwer’s fixed point theorem. Indeed, the claim of lemma A.11 is the existence of a fixed point of the map *P*_*T*_ in *ω*_*T*_(*x*). We show that *ω*_*T*_(*x*) contains an element *y* so that *ω*_*T*_(*y*) is a singleton, say, *ω*_*T*_(*y*)={*z*}. Then *z*∈*ω*_*T*_(*x*) by proposition A.10(c) and *z* is a fixed point by proposition A.10(b).

**A.7. Proof of lemma A.11**

We use the following notation. For *y*∈*Ω*, let

$$\begin{array}{rl} p(y) & :=\inf\omega_{T}(y),\\ q(y) & :=\sup\omega_{T}(y). \end{array}$$

Note that as $\omega_{T}(y)\subseteq \Omega \subset R_{+}^{N}$, *p*(*y*) and *q*(*y*) exist in $R_{+}^{N}$. Moreover, *p*(*y*)≤*q*(*y*) and *ω*_*T*_(*y*) is a singleton if and only if *p*(*y*)=*q*(*y*). The existence of the desired element *y* in *ω*_*T*_(*x*) is established by contradiction. Suppose that no such *y* exists. Denote by *y*_0_ an element in *ω*_*T*_(*x*) that *minimizes* the number of coordinates *j* for which (*p*(*y*))_*j*_ and (*q*(*y*))_*j*_ differ. We then show that there exists *z*_0_∈*ω*_*T*_(*x*) for which *p*(*z*_0_) and *q*(*z*_0_) differ in a smaller number of coordinates than *p*(*y*_0_) and *q*(*y*_0_). Part (c) of lemma A.12 below states a fact that is essential for the construction of *z*_0_. What is also crucial for the proof is the observation that *p*(*y*) and *q*(*y*) are fixed points of *P*_*T*_ which is formulated in lemma A.12(a). Its short proof demonstrates why *monotone dynamical systems admitting a first integral with a positive gradient* are special.

For *y*∈*Ω*, let

A 10 $$J_{y}:=\{j\in \{1,\ldots ,N\}\;|\;{\left(p(y)\right)}_{j}\neq {\left(q(y)\right)}_{j}\},$$

i.e. the set of indices for which (*p*(*y*))_*j*_ and (*q*(*y*))_*j*_ differ.

Lemma A.12 *Let* $f\in F_{T}$. *Then for any* *y*∈*Ω*,

(a) *p*(*y*), *q*(*y*) *are fixed points of* *P*_*T*_.
(b) *P*_*T*_([*p*(*y*),*q*(*y*)])⊆[*p*(*y*),*q*(*y*)].
(c) *If* *p*(*y*)≠*q*(*y*) *then for any* *z*∈*ω*_*T*_(*y*) *there exists a* *j*∈*J*_*y*_ *such that* *z*_*j*_=(*p*(*y*))_*j*_.

To explain property (c), we introduce more notation. For $v,w\in R^{N}$ let

$$\Delta (v,w):=\#\{j\in \{1,\ldots ,N\}\;|\;v_{j}\neq w_{j}\}.$$

Consider the case *p*(*y*)≠*q*(*y*). For any *j*∈{1,…,*N*}∖*J*_*y*_, we have *p*_*j*_(*y*)=*q*_*j*_(*y*), so *z*_*j*_=*p*_*j*_=*q*_*j*_. For any *j*∈*J*_*y*_, we have *p*_*j*_(*y*)<*q*_*j*_(*y*) and property (c) implies that there exists at least one such index such that *p*_*j*_(*y*)=*z*_*j*_. We conclude that

A 11 Δ(z,p(y))<Δ(q(y),p(y)).

Proof of lemma A.12 To simplify the notation, we write from hereon *p*,*q* for *p*(*y*),*q*(*y*). Pick *z*∈*ω*_*T*_(*y*). As *p*≤*z*, theorem A.9 implies that *P*_*T*_(*p*)≤*P*_*T*_(*z*). Thus, *P*_*T*_(*p*)≤*ω*_*T*_(*y*) by proposition A.10(b), and consequently *P*_*T*_(*p*)≤*p*. As *H*(*P*_*T*_(*p*))=*H*(*p*), it follows that *P*_*T*_(*p*)=*p*. The proof that *P*_*T*_(*q*)=*q* proceeds analogously. This proves claim (a). Claim (b) follows directly from (a) and theorem A.9. We prove (c) by contradiction. Assume that there exists *z*∈*ω*_*T*_(*y*) such that *z*_*j*_>*p*_*j*_ for all *j*∈*J*_*y*_. Set

A 12 $$\varepsilon :=\min\{z_{j}-p_{j}\;|\;j\in J_{y}\}.$$

Then *ε*>0. Define

A 13 $$\Gamma :=\left\{x\in [p,q]\;|\;H(x)=H(p)+\frac{\varepsilon }{2}\right\}.$$

Note that for any *j*∈{1,…,*N*}∖*J*_*y*_ we have *p*_*j*_=*q*_*j*_ so if *x*∈*Γ* then *x*_*j*_=*p*_*j*_=*q*_*j*_. The set *Γ* is not empty, as *H*(*q*)≥*H*(*z*)≥*H*(*p*)+*ε*. *Γ* is also compact and convex. Statement (b) and the fact that *H* is a first integral imply that *P*_*T*_(*Γ*)⊆*Γ*. The Brouwer fixed point theorem thus yields the existence of a fixed point of *P*_*T*_ in *Γ*, that is, there exists *x**∈*Γ* such that *P*_*T*_(*x**)=*x**. As *z*∈*ω*_*T*_(*y*) there exists $n^{*}\in N$ such that $|P_{T}^{n^{*}}(y)-z{|}_{1}<\varepsilon /3N$. Define *y** by

$$y_{j}^{*}:=\left\{ \begin{array}{ll} {\left(P_{T}^{n^{*}}(y)\right)}_{j}, & j\in J_{y}\\ z_{j}=p_{j}=q_{j}, & \text{otherwise}. \end{array}\right.$$

As $|P_{T}^{n^{*}}(y)-y^{*}{|}_{1}+|y^{*}-z{|}_{1}\leq |P_{T}^{n^{*}}(y)-z{|}_{1}$, we have $|P_{T}^{n^{*}}(y)-y^{*}{|}_{1}<\varepsilon /3N$ and

A 14 $$|y^{*}-z{|}_{1}<\frac{\varepsilon }{3N}.$$

Observe that

$$x_{j}^{*}=p_{j}=z_{j}=y_{j}^{*}\;\;\text{for}\;j\in \{1,\ldots ,N\}\setminus J_{y}$$

and

$$x_{j}^{*}\leq p_{j}+\frac{\varepsilon }{2}\leq z_{j}-\frac{\varepsilon }{2}<y_{j}^{*}\;\;\text{for}\;j\in J_{y},$$

where the first inequality follows from (A 13), the second from (A 12) and the third from (A 14). Summarizing, *x**≤*y**. As *P*_*T*_(*x**)=*x**, theorem A.9 implies that $x^{*}\leq P_{T}^{k}(y^{*})$ for all *k*≥0. As $\sum_{j}(x_{j}^{*}-p_{j})=\varepsilon /2$, there exists *j*_0_∈{1,…,*N*} such that $x_{j_{0}}^{*}\geq p_{j_{0}}+\varepsilon /2N$. Then ${\left(P_{T}^{k}(y^{*})\right)}_{j_{0}}\geq p_{j_{0}}+\varepsilon /2N$ for all *k*≥0. As *P*_*T*_ is non-expansive by theorem A.8(a), we have in addition $|P_{T}^{k}(y^{*})-P_{T}^{k+n^{*}}(y){|}_{1}\leq |y^{*}-P_{T}^{n^{*}}(y){|}_{1}<\varepsilon /3N$ for all *k*≥0. Hence for all *m*≥*n**,

$${\left(P_{T}^{m}(y)\right)}_{j_{0}}\geq p_{j_{0}}+\frac{\varepsilon }{2N}-\frac{\varepsilon }{3N}=p_{j_{0}}+\frac{\varepsilon }{6N}.$$

We conclude that any *r*∈*ω*_*T*_(*y*) satisfies *r*_*j*_0__≥*p*_*j*_0__+*ε*/6*N*, and thus

$$p_{j_{0}}=\inf\{r_{j_{0}}\;|\;r\in \omega_{T}(y)\}\geq p_{j_{0}}+\frac{\varepsilon }{6N}$$

and this contradiction proves (c). ▪

We can now prove the crucial lemma A.11.

Proof of lemma A.11 As noted above, we need to show that there exists *y*∈*ω*_*T*_(*x*) such that *p*(*y*)=*q*(*y*). To do this, for *y*∈*Ω* let

m(y):=Δ(p(y),q(y))

and for *x*∈*Ω*, let

$$\alpha (x):=\min\{m(y)\;|\;y\in \omega_{T}(x)\}.$$

It suffices to show that *α*(*x*)=0 for all *x*∈*Ω*. We achieve this by contradiction. Assume that there exists *x*∈*Ω* for which *α*(*x*)>0. Then there exists *y*_0_∈*ω*_*T*_(*x*) with *α*(*x*)=*m*(*y*_0_)>0. Let $\tilde{M}:=\max\{\Delta (z,p(y_{0}))\;|\;z\in \omega_{T}(y_{0})\}$. Then (A 11) yields

A 15 $$\tilde{M}<\alpha (x).$$

Choose *z*_0_∈*ω*_*T*_(*y*_0_) such that $\Delta (z_{0},p(y_{0}))=\tilde{M}$. We now show that $m(z_{0})\leq \tilde{M}$. To this end, define

$$J:=\{j\in \{1,\ldots .N\}\;|\;{\left(z_{0}\right)}_{j}>p_{j}(y_{0})\}.$$

Note that $\tilde{M}=\#J$. As *z*_0_∈[*p*(*y*_0_),*q*(*y*_0_)], we have *J*⊆*J*_*y*_0__ and by lemma A.12 we have *J*≠*J*_*y*_0__. Consider the sequence $z(k):=P_{T}^{k}(z_{0})\in \omega_{T}(y_{0})$. By the second statement of theorem A.9, we have *z*_*j*_(*k*)>*p*_*j*_(*y*_0_) for all *j*∈*J* and all k∈N (recall that *p*(*y*_0_) is a fixed point). Hence $\Delta (z(k),p(y_{0}))\geq \#J=\tilde{M}$. As *z*(*k*)∈*ω*_*T*_(*y*_0_), we have by the definition of $\tilde{M}$ also $\Delta (z(k),p(y_{0}))\leq \tilde{M}$ and, therefore, $\Delta (z(k),p(y_{0}))=\tilde{M}$, and

$$J=\{j\in \{1,\ldots ,N\}\;|\;z_{j}(k)>p_{j}(y_{0})\}\;\;\text{for all}\;k\in N.$$

Thus, *z*_*j*_(*k*)=*p*_*j*_(*y*_0_) for all *j*∈{1,…,*N*}∖*J*, so

$$\omega_{T}(z_{0})\subseteq \{v\in R^{N}\;|\;v_{j}=p_{j}(y_{0})\;\text{for all}\;j\in \{1,\ldots ,N\}\setminus J\}.$$

This implies that *p*_*j*_(*z*_0_)=*q*_*j*_(*z*_0_) for all *j*∈{1,…,*N*}∖*J*, and thus $m(z_{0})=\Delta (p(z_{0}),q(z_{0}))\leq \#J=\tilde{M}$. Combining this with the fact that *z*_0_∈*ω*_*T*_(*y*_0_)⊆*ω*_*T*_(*x*) and (A 15) yields

$$\alpha (x)\leq m(z_{0})\leq \tilde{M}<\alpha (x)$$

and this contradiction completes the proof of lemma A.11. ▪
